# Causal relationship between immune cells and heart failure: A Mendelian randomization study

**DOI:** 10.1097/MD.0000000000041247

**Published:** 2025-01-10

**Authors:** Shenghua Lu, Yunfeng Yu, Zheqin Zhu, Min Wang, Rongzhen Liu, Jianhe Liu

**Affiliations:** a The First Clinical College of Chinese Medicine, Hunan University of Chinese Medicine, Changsha, Hunan, China; b Branch of National Clinical Research Center for Chinese Medicine Cardiology, The First Hospital of Hunan University of Chinese Medicine, Changsha, Hunan, China.

**Keywords:** genetic susceptibility, heart failure, immune cells, Mendelian randomization, phenotypes

## Abstract

This study aimed to evaluate the causal effects of different immune cells on heart failure (HF) using Mendelian randomization (MR). Datasets for immune cell phenotypes and HF were obtained from European Bioinformatics Institute and FinnGen. Then, single nucleotide polymorphisms were screened according to the basic assumptions of MR. Subsequently, inverse variance weighted was used as primary tool for MR analysis, and Cochran Q and leave-one-out analyses were used to assess heterogeneity and robustness, respectively. MR analysis showed that cluster of differentiation (CD) 66b++ myeloid cell absolute count (AC) (odds ratio [OR] 1.043, 95% confidence interval [CI] 1.001–1.088, *P* = .045), human leukocyte antigen D-related on CD14– CD16+ monocyte (OR 1.030, 95% CI 1.005–1.056, *P* = .019), IgD on unsw mem (OR 1.046, 95% CI 1.015–1.078, *P* = .003), CD4 on CD4+ (OR 1.039, 95% CI 1.009–1.070, *P* = .011), CD24 on IgD+ CD38– (OR 1.026, 95% CI 1.000–1.052, *P* = .046), CD20 on CD24 + CD27+ (OR 1.032, 95% CI 1.003–1.061, *P* = .029), CD19 on CD20– (OR 1.037, 95% CI 1.005–1.071, *P* = .023), CD62L– CD86 + myeloid dendritic cell %DC (OR 1.032, 95% CI 1.004–1.061, *P* = .027), human leukocyte antigen D-related + CD4 + AC (OR 1.037, 95% CI 1.003–1.072, *P* = .032), and effector memory CD8br AC (OR 1.048, 95% CI 1.021–1.076, *P* < .001) were associated with increased genetic susceptibility to HF. Cochran Q and sensitivity analyses showed that the results had no heterogeneity and were robust. This MR analysis revealed 10 immune cell phenotypes associated with increased genetic susceptibility to HF. These findings provide new directions for understanding the pathogenesis of HF and developing novel therapies.

## 1. Introduction

Heart failure (HF) is a complex syndrome characterized by impairment of the heart’s contractile and/or diastolic function due to various factors, resulting in insufficient cardiac output to meet the body’s basic metabolic needs.^[1]^ It is mainly manifested as dyspnea, activity limitation, and tissue edema, and is characterized by a high incidence, high mortality, and poor prognosis.^[2]^ Epidemiological surveys showed that the number of HF patients worldwide was 33.5 million in 1990. However, by 2017, the number of HF patients had increased to 64.3 million.^[3]^ With the increase in population and the exacerbation of the aging problem, the number of HF patients globally may further increase in the future.^[3]^ Although the pathogenesis of HF has not been fully elucidated, it is generally believed to be related to factors such as myocardial metabolic abnormalities, sympathetic overactivation, cardiac remodeling, and overactivation of the renin–angiotensin–aldosterone system.^[2]^ Despite the series of preventive and treatment measures taken by modern medicine based on these pathogenic mechanisms, the incidence and mortality of HF remain high, and the prognosis is poor.^[4,5]^ Therefore, we need to understand and identify the pathogenesis of HF from different perspectives and find treatment strategies for HF through more diverse approaches.

The immune system plays a crucial role in cardiovascular diseases, and abnormal activation of immune cells and excessive release of inflammatory factors can lead to severe myocardial damage and pump failure.^[6–8]^ Recent studies have shown that T lymphocytes, B lymphocytes, monocytes, and macrophages are involved in myocardial remodeling and the occurrence of HF.^[9]^ Studies have found that compared to healthy individuals, HF patients exhibited impaired T cell reactivity, with a significantly higher proportion of cluster of differentiation (CD) 4 + T cells.^[10,11]^ Through selective inhibition of CD4 + T cells, the pathological cardiac remodeling in heart failure (HF) patients can be attenuated.^[12]^ These pieces of evidence suggest that immune cells may play an important role in HF. Therefore, it is necessary for us to comprehensively evaluate the causal effects of different immune cells on HF using effective and comprehensive methods.

Mendelian randomization (MR) is a method for evaluating the effect of exposure on outcomes through genetic variation.^[13]^ Compared to traditional research methods, it reduces potential biases from confounding factors and reverse causation.^[14]^ Therefore, MR can provide effective evidence for the correlation analysis between different immune cells and diseases.^[15]^ In this study, we utilized MR to analyze the effects of 731 immune cell phenotypes on genetic susceptibility to HF, aiming to reveal the immune cell phenotypes associated with HF.

## 2. Materials and methods

### 2.1. Study design

**Figure 1.**
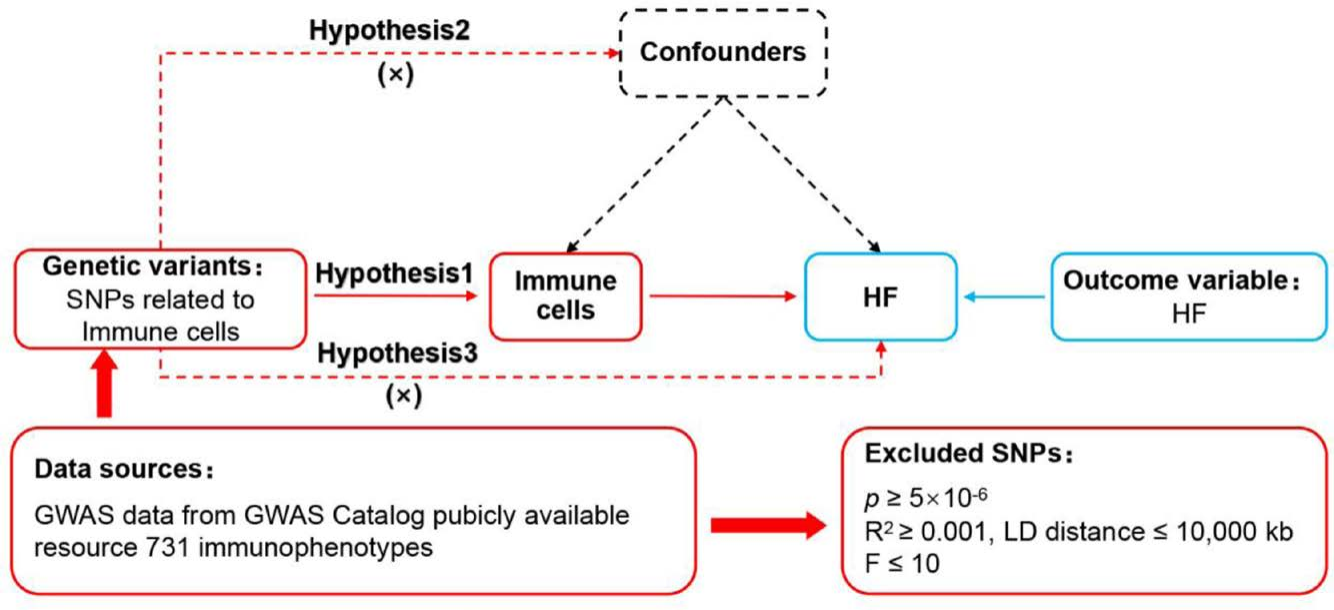
MR design for immune cells on genetic susceptibility to HF. HF = heart failure; MR = Mendelian randomization.

MR was based on the assumptions of relevance, independence, and exclusivity.^[16]^ Among them, the relevance assumption required that single nucleotide polymorphisms (SNPs) were closely related to exposure, the independence assumption required that SNPs were independent of confounding factors, and the exclusivity assumption stated that SNPs could not affect the outcome through nonexposure pathways. The study design is shown in Figure 1.

### 2.2. Data sources

We obtained immune cell data, numbered from GCST0001391 to GCST0002121, from the European Bioinformatics Institute.^[17]^ This dataset contains 731 immune cells, covering Treg cells, mature T cells, B cells, natural killer cells, monocytes, myeloid cells, and others. Additionally, we obtained the HF dataset, numbered finngen_R10_I9_HEARTFAIL_EXMORE, from FinnGen (www.finngen.fi/fi), which contained genetic information from 387,444 Europeans. Given that these databases are publicly available, this study did not require additional ethical approval.

### 2.3. Selection of genetic instrumental variables

First, we limited *P* < 5 × 10^−6^ to search for SNPs closely related to each immune cell phenotype to satisfy the relevance assumption. Second, we further limited *R*² < 0.001 and kb = 10,000 to exclude biases associated with linkage disequilibrium. Third, we limited F > 10 to ensure that SNPs were strongly correlated. F = [*R*^2^/(1 − *R*^2^)] × [(N − K − 1)/K]. *R*² represents the cumulative explanatory variance of selected instrumental variables for exposure, N represents the total sample size of the genome association study, and K represents the number of paired samples. Fourth, we excluded confounding factors that may affect the results through PhenoScanner to satisfy the independence assumption. Fifth, we excluded mismatched SNPs based on allele frequency when aligning allele directions. Finally, we used the MR-Pleiotropy Residual Sum and Outlier method to remove outlier SNPs (*P* < 1).

### 2.4. Data analysis

This study followed the Strengthening the Reporting of Observational Studies in Epidemiology-MR reporting guidelines,^[18]^ and all analyses and data visualization were completed using R 4.3.1 software. Inverse variance weighted, a method that can achieve unbiased causal analysis, was set as the primary tool for MR analysis. Weighted median, a method less sensitive to error values and outliers, and MR-Egger, a method that provides effective analysis in the presence of pleiotropy, were set as secondary tools for MR analysis. Subsequently, the MR-Egger intercept was used to assess horizontal pleiotropy, with *P* ≥ .05 indicating no significant horizontal pleiotropy, satisfying the exclusion restriction assumption. Cochran Q was used to evaluate heterogeneity, with *P* ≥ .05 indicating no significant heterogeneity. Leave-one-out sensitivity analysis was used to assess robustness, with combined effect sizes on the same side indicating robust results.

## 3. Results

### 3.1. Genetic instrumental variables

After screening through the aforementioned 6 steps, SNPs that met the basic assumptions were included in the MR analysis. MR analysis reported that 10 immune cell phenotypes were significantly associated with increased genetic susceptibility to HF, and the relevant SNPs are shown in Table S1 to S10, Supplemental Digital Content, http://links.lww.com/MD/O267.

### 3.2. Two-sample MR analysis

**Figure 2.**
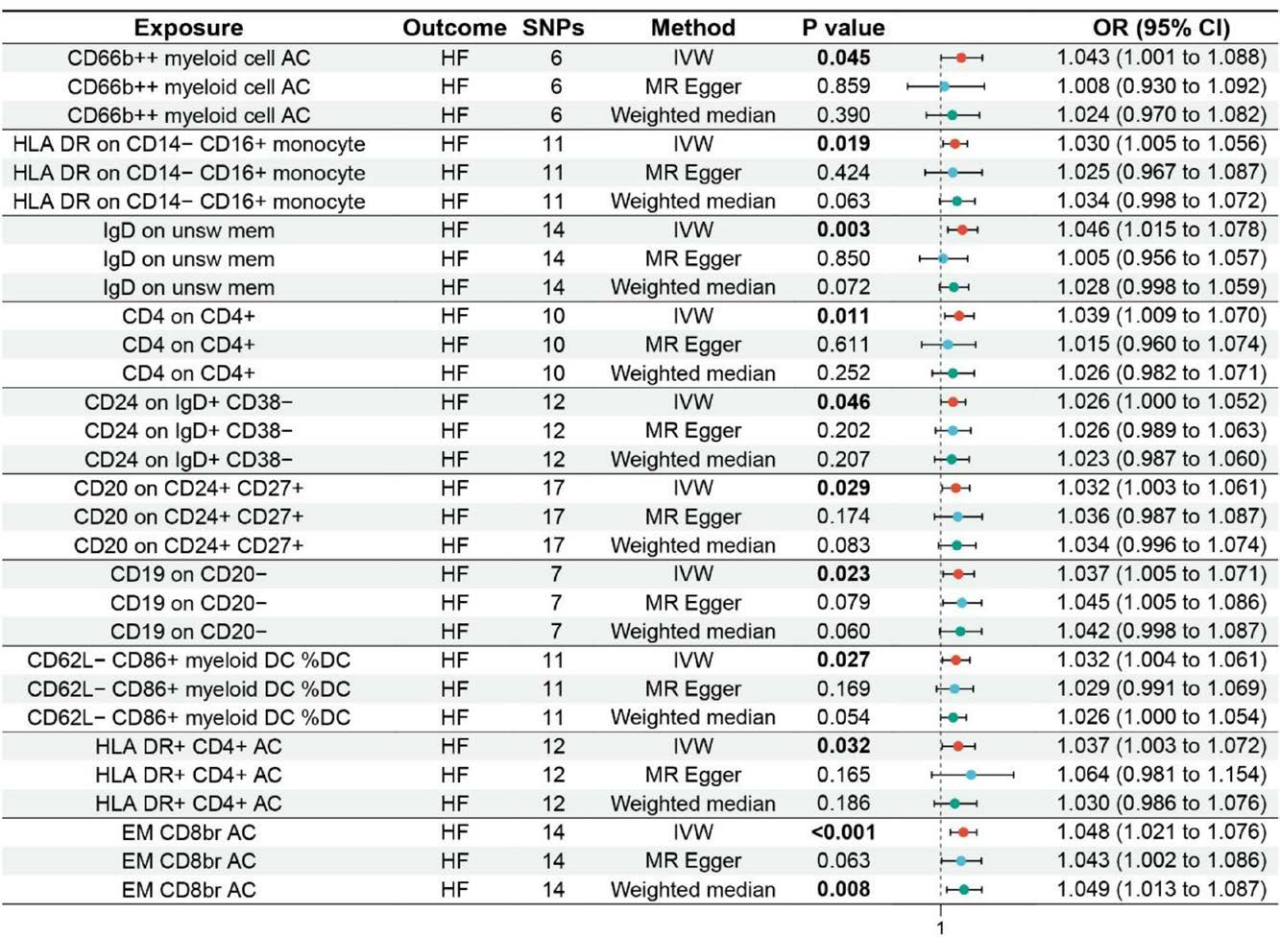
Forest plot of MR analysis for immune cells on genetic susceptibility to HF. AC = absolute count; CI = confidence interval; CD = cluster of differentiation; DC = dendritic cell; EM = effector memory; HF = heart failure; HLA DR = human leukocyte antigen D-related; MR = Mendelian randomization; IVW = inverse variance weighted.

The MR analysis showed that CD66b++ myeloid cell absolute count (AC) (odds ratio [OR] 1.043, 95% confidence interval [CI] 1.001–1.088, *P* = .045), human leukocyte antigen D-related (HLA DR) on CD14− CD16 + monocyte (OR 1.030, 95% CI 1.005–1.056, *P* = .019), IgD on unsw mem (OR 1.046, 95% CI 1.015–1.078, *P* = .003), CD4 on CD4 + (OR 1.039, 95% CI 1.009–1.070, *P* = .011), CD24 on IgD + CD38− (OR 1.026, 95% CI 1.000–1.052, *P* = .046), CD20 on CD24 + CD27 + (OR 1.032, 95% CI 1.003–1.061, *P* = .029), CD19 on CD20− (OR 1.037, 95% CI 1.005–1.071, *P* = .023), CD62L− CD86 + myeloid dendritic cell (DC) %DC (OR 1.032, 95% CI 1.004–1.061, *P* = .027), HLA DR + CD4 + AC (OR 1.037, 95% CI 1.003–1.072, *P* = .032), and effector memory (EM) CD8br AC (OR 1.048, 95% CI 1.021–1.076, *P* < .001) were associated with increased genetic susceptibility to HF. The forest plot and scatter plot are shown in Figure 2 and Figure S1, Supplemental Digital Content, http://links.lww.com/MD/O267, respectively. The MR-Egger intercept indicated no horizontal pleiotropy in the results (*P* ≥ .05), see Table S11, Supplemental Digital Content, http://links.lww.com/MD/O267.

### 3.3. Heterogeneity and sensitivity analysis

Cochran Q showed no heterogeneity in the MR analysis results (*P* ≥ .05), see Table S12, Supplemental Digital Content, http://links.lww.com/MD/O267 and Figure S2, Supplemental Digital Content, http://links.lww.com/MD/O267. Sensitivity analysis demonstrated that these results were robust, see Figure S3, Supplemental Digital Content, http://links.lww.com/MD/O267.

## 4. Discussion

HF is the middle and late stage of various cardiovascular diseases and one of the main causes of death in patients with cardiovascular diseases.^[1]^ Although the role of immune cells in HF has attracted increasing attention in recent years, the effects of different immune cell phenotypes on HF have not been fully elucidated. To our knowledge, this is the first MR analysis to evaluate the effects of 731 immune cell phenotypes on genetic susceptibility to HF, providing genetic insights into the causal relationship between different immune cells and HF. These findings suggest that CD66b++ myeloid cell AC, HLA DR on CD14− CD16 + monocyte, IgD on unsw mem, CD4 on CD4+, CD24 on IgD + CD38−, CD20 on CD24 + CD27+, CD19 on CD20−, CD62L− CD86 + myeloid DC %DC, HLA DR + CD4 + AC, and EM CD8br AC are associated with increased genetic susceptibility to HF.

Recently, Li et al^[19]^ published an MR study evaluating the causal relationship between immune cells and all-cause HF. However, their study primarily focused on assessing congestive HF, which differed from the systolic HF included in our study. Despite the differences in participants between the 2 studies, both revealed the potential role of immune cells in HF. The 2 studies employed similar methodological designs, incorporating large-scale GWAS summary data, which lends credibility to their findings and allowed for mutual corroboration and complementarity. It is noteworthy that both studies reported an association between CD62L-CD86 + myeloid DC %DC and increased risk of HF, emphasizing the potential role of memory T cells, particularly CD8 + T cells, in the pathogenesis of HF. However, Li et al^[19]^ also reported associations between DC AC, CD62L-CD86 + myeloid DC AC, CD39 + CD8 + T cell% CD8 + T cell, and CD3 on central memory CD4 + T cell with increased risk of HF, which differed from our findings. This discrepancy may stem from the outcome datasets, as Li et al’s^[19]^ study primarily focused on congestive HF rather than the systolic HF that our study concentrated on. Although the immune cell phenotypes reported in the 2 studies were not entirely identical, both confirmed the potential role of immune cells in different types of HF.

CD66b++ myeloid cells refer to myeloid cells that are strongly positive for CD66b, mainly including activated neutrophils.^[20]^ Studies have found that neutrophil CD66b is significantly positively correlated with N-terminal pro-B-type natriuretic peptide, indicators that measure the severity of HF.^[21]^ Another study found that in elderly HF patients, the proportion of neutrophils was associated with an increase in major cardiovascular events.^[22]^ These pieces of evidence suggest that CD66b++ myeloid cells are associated with a poor prognosis of HF, indicating that they are potential risk factors for HF.

HLA DR on CD14− CD16 + monocyte refers to nonclassical monocytes that are negative for CD14, positive for CD16, and express HLA DR on their surface. HLA-DR is a human class II major histocompatibility complex antigen that is constitutively expressed on the surface of B lymphocytes, monocytes, and macrophages, and participates in antigen presentation to CD4 + T cells.^[23]^ Research has found that the tissue-resident CXCL8^hi^CCR2^+^HLA-DR^hi^ macrophage subset may promote leukocyte infiltration by interacting with endothelial cells, exacerbating myocardial inflammation and fibrosis in HF patients.^[24]^ High expression of HLA-DR is one of the hallmarks of this special macrophage subset, suggesting that it may have stronger antigen-presenting capabilities and play an important role in immune imbalance in HF. Under steady-state conditions, nonclassical monocytes patrol within blood vessels through recognition and clearance of apoptotic endothelial cells.^[25,26]^ However, in pathological conditions, these cells may contribute to disease progression. Coronary artery disease, the most common cause of HF, has been associated with altered monocyte function. Urbanski et al^[27]^ demonstrated that elevated levels of nonclassical monocytes in coronary artery disease patients strongly correlate with late-stage vascular dysfunction and vascular oxidative stress. Furthermore, Hilgendorf et al^[28]^ found that this monocyte subset promotes angiogenesis and fibroblast accumulation during the late phase of myocardial ischemia–reperfusion. These findings suggest that HLA DR on CD14− CD16 + monocytes may contribute to HF pathogenesis through modulation of immune and inflammatory responses.

CD4 on CD4 + refers to CD4 + T cells expressing CD4 on their surface. CD4 is a transmembrane glycoprotein receptor on the surface of T lymphocytes that plays a crucial role in myocardial remodeling.^[29]^ Studies have found that a considerable number of CD4 + T cells infiltrate the fibrotic myocardium of HF patients, suggesting the presence of persistent immune activation in the body.^[24]^ Targeting the specificity of CD4 + T cells can reduce T cell receptor activation and improve left ventricular remodeling and progressive HF in HF mice.^[12]^ It can be seen that CD4 on CD4 + is a potential risk factor for HF, and targeted inhibition of CD4 on CD4 + may be a potential strategy for the treatment of HF in the future.

CD24 on IgD + CD38− refers to immature B cells expressing CD24 and IgD on their surface and not expressing CD38. Although there are no reports on the relationship between CD24 and HF, existing studies support the association of CD24 with risk factors for HF, such as hypertension and coronary heart disease.^[30]^ Studies have found that CD24 may be involved in the process of hypertension-related vascular endothelial injury, changes in permeability, and kidney damage by affecting vascular endothelial function.^[31]^ Another study found that high-density lipoprotein cholesterol values were inversely proportional to immature B cells expressing IgD on the cell surface.^[32]^ Given that high levels of high-density lipoprotein cholesterol are protective factors for coronary heart disease and atherosclerosis,^[33]^ immature B cells expressing IgD on the cell surface may be potential risk factors for coronary heart disease and HF. In summary, these pieces of evidence suggest that CD24 on IgD + CD38− may be a potential risk factor for HF.

CD20 on CD24 + CD27 + refers to mature B cells expressing CD20, CD24, and CD27 on their surface. Rituximab is a CD20-targeted monoclonal antibody. Ma et al^[34]^ found that administration of rituximab significantly improved cardiac function and inhibited ventricular dilation, myocyte hypertrophy, fibrosis, and oxidative stress. This suggests that CD20 is a potential risk factor for HF and that anti-CD20 therapy is an effective method for treating HF. Furthermore, in vitro studies have shown that the interaction between CD27 on B cells and its co-stimulatory ligand CD70 mediates the differentiation of B cells into plasma cells.^[35]^ However, there is a negative correlation between B cell depletion and the formation of atherosclerotic plaques in the body.^[36]^ This implies that CD27 on B cells may increase the risk of coronary artery disease by promoting atherosclerotic immune responses, thereby increasing the potential risk of HF. These pieces of evidence suggest that CD20, CD24, and CD27 may be associated with increased HF risk, indicating that CD20 on CD24 + CD27 + may be a risk factor for HF.

CD19 on CD20− refers to B cells expressing CD19 but not CD20. Guo et al^[37]^ found an increased percentage of circulating CD19 + Bregs in the peripheral blood of HF patients. Yu et al^[38]^ analyzed the peripheral blood of 56 patients with dilated nonischemic cardiomyopathy by flow cytometry and found that the frequency of CD19 + B cells was higher in HF patients compared to healthy subjects, and CD19 + B cells were actively replicating. Although no evidence supports a direct relationship between CD19 on CD20− and HF, some studies have confirmed its role in autoimmune diseases. Relevant studies have found that the proportion of CD19 on CD20− B cells is significantly higher in patients with systemic lupus erythematosus and multiple sclerosis and exhibits an activated phenotype.^[39,40]^ This suggests that CD19 on CD20− B cells may produce a large number of autoantibodies through overactivation, which then form immune complexes deposited in myocardial tissues, leading to myocardial damage.

CD62L− CD86 + myeloid DC %DC refers to the proportion of myeloid DC that do not express CD62L and express CD86 on their surface among DC. CD86 is an important co-stimulatory molecule that is significantly upregulated in the peripheral blood of atherosclerotic patients and in DC induced by oxidized low-density lipoprotein.^[41,42]^ Coronary heart disease is the leading cause of HF in many patients and is associated with long-term hypercholesterolemia and atherosclerosis.^[43]^ Therefore, CD86 may indirectly increase the risk of HF by increasing the risk of atherosclerosis, suggesting that CD62L− CD86 + myeloid DC %DC is a potential risk factor for HF.

HLA DR + CD4 + AC refers to the AC of T lymphocytes expressing HLA DR and CD4 on their surface. CD4 + T cell responses restricted by HLA-DR alleles are stronger than those restricted by HLA-DQ and HLA-DP alleles and thus possess stronger immune response capabilities.^[44]^ Previous views believed that decreased T cell activation was associated with decreased subclinical atherosclerosis.^[45]^ This implies that HLA-DR CD4 + T cells with stronger immune activity can promote atherosclerosis, thereby inducing and promoting HF. Subsequent studies found that HLA-DR + CD38-CD4 + T cells, an atherosclerosis inflammation related marker, were significantly reduced in dyslipidemia patients who received pitavastatin calcium treatment.^[46]^ These pieces of evidence suggest that HLA DR + CD4 + AC is associated with an increased risk of atherosclerosis, hinting that it is an indirect risk factor for HF.

EM CD8br AC refers to the AC of EM T lymphocytes with high CD8 expression on their surface. A research has showed that CD8 + T cells in the heart are associated with the transformation of resident and infiltrating macrophages into cardioprotective macrophages, which can lead to destructive cardiac myocyte hypertrophy.^[47]^ In a mouse model of myocardial infarction, CD8 + T lymphocytes were recruited and activated in ischemic heart tissue and released granzyme B, leading to cardiomyocyte apoptosis, adverse ventricular remodeling, and deterioration of myocardial function.^[48]^ Subsequent studies found that depletion of CD8 + T cells reduced apoptosis and inflammatory responses in the ischemic myocardium, thereby reducing myocardial injury and improving cardiac function.^[48]^ Given that myocardial infarction is a common situation leading to HF, targeted blocking of EM CD8br AC may be a potential treatment strategy for HF.

IgD on unsw mem refers to immature memory B cells expressing IgD on their surface. Although current literature supports the notion that B cell depletion is associated with reduced infarct size and alleviated adverse ventricular remodeling,^[49–51]^ there is a lack of direct evidence linking immature memory B cells to HF. Therefore, future clinical studies are needed to explore the expression levels and roles of IgD on unsw mem in HF patients.

Our study faced some inherent limitations when exploring the relationship between immune cells and HF. First, this MR analysis was conducted based on European populations, so further validation is needed to determine whether similar results exist in other populations. Second, due to the lack of HF grading information in the database, we could not evaluate the role of immune cells at different stages of HF through subgroup analysis. Third, while this study reported 10 immune cell phenotypes associated with HF, relying solely on statistical effects does not explain their biological mechanisms in HF. Therefore, we look forward to future researchers continuously improving datasets from different races to strive for equal health across races. Additionally, researchers should promote the development of molecular biology experiments and clinical trials to reveal the mechanisms of action of immune cells in HF.

## 5. Conclusion

This MR analysis suggests that CD66b++ myeloid cell AC, HLA DR on CD14- CD16 + monocyte, IgD on unsw mem, CD4 on CD4+, CD24 on IgD + CD38-, CD20 on CD24 + CD27+, CD19 on CD20-, CD62L- CD86 + myeloid DC %DC, HLA DR + CD4 + AC, and EM CD8br AC are associated with increased genetic susceptibility to HF. This findings provide new directions for understanding the pathogenesis of HF and developing novel therapies. However, due to limited clinical evidence, more biological studies are needed in the future to elucidate the specific mechanisms and effects of immune cell phenotypes on HF.

## Author contributions

**Conceptualization:** Shenghua Lu, Yunfeng Yu.

**Data curation:** Shenghua Lu, Yunfeng Yu.

**Formal analysis:** Shenghua Lu, Yunfeng Yu.

**Methodology:** Shenghua Lu, Yunfeng Yu.

**Software:** Yunfeng Yu, Zheqin Zhu, Min Wang.

**Supervision:** Zheqin Zhu, Min Wang, Rongzhen Liu.

**Writing – original draft:** Shenghua Lu, Yunfeng Yu.

**Writing – review & editing:** Jianhe Liu.

## Supplementary Material


