# Supplementary material for: Causal relationship between immune cells and heart failure: A Mendelian randomization study
Source: Medicine (Baltimore). 2025 Jan 10;104(2):e41247. doi: 10.1097/MD.0000000000041247 (PMC11730111; doi:10.1097/MD.0000000000041247)
Supplement: Supplementary file 1 [file medi-104-e41247-s001.docx]

**Supplementary Table S1** Information of identified SNPs in exposure (CD66b++ myeloid cell AC) and outcomes (HF).

|  |  | | | **Exposure (CD66b++ myeloid cell AC)** | | |  | **Outcome (HF)** | | | | |
| --- | --- | --- | --- | --- | --- | --- | --- | --- | --- | --- | --- | --- |
|  | **SNP** | **EA** | **OA** | **β** | **SE** | ***p* value** |  | **Case** | **Control** | **β** | **SE** | ***p* value** |
| 1 | rs11218704 | G | A | 0.3401 | 0.06951 | 1.08E-06 |  | 29,672 | 357,772 | 0.0172467 | 0.0178256 | 0.333283 |
| 2 | rs114469087 | A | T | 0.4988 | 0.1035 | 1.54E-06 |  | 29,672 | 357,772 | 0.0118224 | 0.0374053 | 0.751957 |
| 3 | rs1899009 | G | A | 0.2036 | 0.04022 | 4.56E-07 |  | 29,672 | 357,772 | 0.00259822 | 0.0107686 | 0.809341 |
| 4 | rs2070901 | T | G | 0.189 | 0.03644 | 2.37E-07 |  | 29,672 | 357,772 | 0.0224459 | 0.010433 | 0.031442 |
| 5 | rs2630166 | G | C | 0.1999 | 0.04232 | 2.50E-06 |  | 29,672 | 357,772 | 0.0104231 | 0.0103515 | 0.313976 |
| 6 | rs72845259 | A | G | 1.74 | 0.3719 | 3.10E-06 |  | 29,672 | 357,772 | 0.0252913 | 0.0693771 | 0.715448 |

SNP, single nucleotide polymorphism; CD, cluster of differentiation; AC, absolute count; HF, heart failure; EA, effect allele; OA, other allele; SE, standard error.

**Supplementary Table S2** Information of identified SNPs in exposure (HLA DR on CD14- CD16+ monocyte) and outcomes (HF).

|  |  | | | **Exposure (HLA DR on CD14- CD16+ monocyte)** | | |  | **Outcome (HF)** | | | | |
| --- | --- | --- | --- | --- | --- | --- | --- | --- | --- | --- | --- | --- |
|  | **SNP** | **EA** | **OA** | **β** | **SE** | ***p* value** |  | **Case** | **Control** | **β** | **SE** | ***p* value** |
| 1 | rs116671810 | A | G | 0.3239 | 0.05515 | 4.66E-09 |  | 29,672 | 357,772 | 0.132752 | 0.159131 | 0.404151 |
| 2 | rs13076004 | G | A | -0.1591 | 0.03462 | 4.47E-06 |  | 29,672 | 357,772 | -0.0195091 | 0.0113924 | 0.08681 |
| 3 | rs2297606 | T | G | -0.1374 | 0.02879 | 1.87E-06 |  | 29,672 | 357,772 | -0.00364475 | 0.00987467 | 0.712052 |
| 4 | rs56353032 | T | A | -0.175 | 0.03586 | 1.11E-06 |  | 29,672 | 357,772 | -0.0123035 | 0.0107329 | 0.251659 |
| 5 | rs61742525 | A | G | 0.313 | 0.04821 | 9.64E-11 |  | 29,672 | 357,772 | 0.0503351 | 0.0726132 | 0.488187 |
| 6 | rs6808893 | T | C | -0.2043 | 0.02491 | 3.27E-16 |  | 29,672 | 357,772 | 0.00464672 | 0.00941898 | 0.621776 |
| 7 | rs6917212 | G | C | -0.476 | 0.03201 | 1.32E-48 |  | 29,672 | 357,772 | -0.0165319 | 0.0101662 | 0.103914 |
| 8 | rs73499473 | T | C | 0.3801 | 0.02898 | 1.91E-38 |  | 29,672 | 357,772 | -0.00371798 | 0.0105584 | 0.724738 |
| 9 | rs74970695 | A | T | 0.2118 | 0.04539 | 3.19E-06 |  | 29,672 | 357,772 | -0.0114439 | 0.0167383 | 0.49417 |
| 10 | rs8057874 | C | G | 0.1477 | 0.02631 | 2.11E-08 |  | 29,672 | 357,772 | 0.00465187 | 0.00949509 | 0.624187 |
| 11 | rs9269109 | C | T | 0.3511 | 0.02921 | 1.19E-32 |  | 29,672 | 357,772 | 0.0232748 | 0.00998654 | 0.0197733 |

SNP, single nucleotide polymorphism; HLA DR, human leukocyte antigen D-related; CD, cluster of differentiation; HF, heart failure; EA, effect allele; OA, other allele; SE, standard error.

**Supplementary Table S3** Information of identified SNPs in exposure (IgD on unsw mem) and outcomes (HF).

|  |  | | | **Exposure (IgD on unsw mem)** | | |  | **Outcome (HF)** | | | | |
| --- | --- | --- | --- | --- | --- | --- | --- | --- | --- | --- | --- | --- |
|  | **SNP** | **EA** | **OA** | **β** | **SE** | ***p* value** |  | **Case** | **Control** | **β** | **SE** | ***p* value** |
| 1 | rs11014404 | G | A | 0.1275 | 0.02747 | 3.60E-06 |  | 29,672 | 357,772 | 0.00394862 | 0.0095298 | 0.678622 |
| 2 | rs1181899 | C | T | 0.1892 | 0.04091 | 3.88E-06 |  | 29,672 | 357,772 | 0.0424997 | 0.0118168 | 0.000322456 |
| 3 | rs12609856 | A | C | 0.1162 | 0.02519 | 4.12E-06 |  | 29,672 | 357,772 | 0.00898318 | 0.00937114 | 0.33776 |
| 4 | rs139084170 | A | C | 0.3794 | 0.05758 | 5.09E-11 |  | 29,672 | 357,772 | -0.0364485 | 0.0495746 | 0.462202 |
| 5 | rs147653204 | A | G | -0.4436 | 0.08543 | 2.18E-07 |  | 29,672 | 357,772 | -0.0234563 | 0.121251 | 0.846605 |
| 6 | rs28723652 | A | C | 0.4108 | 0.07319 | 2.15E-08 |  | 29,672 | 357,772 | 0.0163307 | 0.0146207 | 0.26401 |
| 7 | rs55861228 | C | T | 0.247 | 0.05051 | 1.05E-06 |  | 29,672 | 357,772 | 0.051877 | 0.0204975 | 0.0113773 |
| 8 | rs709589 | T | C | -0.5907 | 0.0302 | 4.31E-81 |  | 29,672 | 357,772 | -0.0140193 | 0.0102671 | 0.172106 |
| 9 | rs73022545 | A | G | 0.244 | 0.05311 | 4.48E-06 |  | 29,672 | 357,772 | 0.0106151 | 0.02995 | 0.723019 |
| 10 | rs75544266 | T | C | 0.3082 | 0.0621 | 7.22E-07 |  | 29,672 | 357,772 | -0.00992415 | 0.0257646 | 0.7001 |
| 11 | rs76784736 | A | T | -0.2696 | 0.05839 | 4.04E-06 |  | 29,672 | 357,772 | -0.0357065 | 0.0325349 | 0.272429 |
| 12 | rs9267517 | A | G | -0.2671 | 0.03579 | 1.06E-13 |  | 29,672 | 357,772 | -0.0283779 | 0.0588113 | 0.629432 |
| 13 | rs9276702 | G | A | 0.2205 | 0.03527 | 4.55E-10 |  | 29,672 | 357,772 | 0.0212145 | 0.0156535 | 0.175338 |
| 14 | rs9923814 | C | T | 0.1694 | 0.03333 | 3.94E-07 |  | 29,672 | 357,772 | 0.00946211 | 0.0108927 | 0.385032 |

SNP, single nucleotide polymorphism; HF, heart failure; EA, effect allele; OA, other allele; SE, standard error.

**Supplementary Table S4** Information of identified SNPs in exposure (CD4 on CD4+) and outcomes (HF).

|  |  | | | **Exposure (CD4 on CD4+)** | | |  | **Outcome (HF)** | | | | |
| --- | --- | --- | --- | --- | --- | --- | --- | --- | --- | --- | --- | --- |
|  | **SNP** | **EA** | **OA** | **β** | **SE** | ***p* value** |  | **Case** | **Control** | **β** | **SE** | ***p* value** |
| 1 | rs11587381 | G | C | -0.3266 | 0.06286 | 2.18E-07 |  | 29,672 | 357,772 | -0.0304112 | 0.01406 | 0.0305443 |
| 2 | rs11615628 | A | G | -0.2753 | 0.03163 | 5.31E-18 |  | 29,672 | 357,772 | 0.00361211 | 0.0103147 | 0.726196 |
| 3 | rs118120903 | T | C | -0.3906 | 0.08422 | 3.67E-06 |  | 29,672 | 357,772 | -0.0204301 | 0.0507673 | 0.687371 |
| 4 | rs1833936 | C | G | 0.1379 | 0.03012 | 4.87E-06 |  | 29,672 | 357,772 | -0.00116314 | 0.0101511 | 0.908776 |
| 5 | rs3129267 | G | C | -0.1877 | 0.03439 | 5.22E-08 |  | 29,672 | 357,772 | -0.0208457 | 0.00979875 | 0.0333887 |
| 6 | rs4959028 | G | A | -0.1654 | 0.03542 | 3.15E-06 |  | 29,672 | 357,772 | -0.0251576 | 0.0150113 | 0.0937562 |
| 7 | rs6068656 | T | C | -0.3227 | 0.06884 | 2.89E-06 |  | 29,672 | 357,772 | -0.00076285 | 0.014527 | 0.95812 |
| 8 | rs61951572 | C | T | 0.3853 | 0.08181 | 2.59E-06 |  | 29,672 | 357,772 | 0.0140634 | 0.0247179 | 0.569386 |
| 9 | rs62027494 | G | C | -1.128 | 0.2427 | 3.49E-06 |  | 29,672 | 357,772 | -0.0280206 | 0.0300375 | 0.350895 |
| 10 | rs6580816 | T | C | 0.1587 | 0.03001 | 1.32E-07 |  | 29,672 | 357,772 | 0.0122899 | 0.00944233 | 0.193063 |

SNP, single nucleotide polymorphism; CD, cluster of differentiation; HF, heart failure; EA, effect allele; OA, other allele; SE, standard error.

**Supplementary Table S5** Information of identified SNPs in exposure (CD24 on IgD+ CD38-) and outcomes (HF).

|  |  | | | **Exposure (CD24 on IgD+ CD38-)** | | |  | **Outcome (HF)** | | | | |
| --- | --- | --- | --- | --- | --- | --- | --- | --- | --- | --- | --- | --- |
|  | **SNP** | **EA** | **OA** | **β** | **SE** | ***p* value** |  | **Case** | **Control** | **β** | **SE** | ***p* value** |
| 1 | rs1007713 | A | G | 0.1897 | 0.04144 | 4.85E-06 |  | 29,672 | 357,772 | 0.0267617 | 0.0108519 | 0.01366 |
| 2 | rs11781090 | T | C | 0.1316 | 0.02643 | 6.71E-07 |  | 29,672 | 357,772 | 0.0115831 | 0.00944493 | 0.220053 |
| 3 | rs138034828 | C | G | 0.2825 | 0.05859 | 1.49E-06 |  | 29,672 | 357,772 | 0.00773352 | 0.0319774 | 0.808902 |
| 4 | rs143053604 | G | A | -0.8464 | 0.1643 | 2.70E-07 |  | 29,672 | 357,772 | -0.0589257 | 0.0361651 | 0.103238 |
| 5 | rs144378737 | G | A | -2.105 | 0.4528 | 3.47E-06 |  | 29,672 | 357,772 | -0.0479884 | 0.035773 | 0.179768 |
| 6 | rs2236073 | A | G | 0.1396 | 0.02534 | 3.86E-08 |  | 29,672 | 357,772 | -0.00999621 | 0.00936064 | 0.285566 |
| 7 | rs77636548 | C | T | -0.3484 | 0.06738 | 2.45E-07 |  | 29,672 | 357,772 | -0.00713717 | 0.051085 | 0.888888 |
| 8 | rs783418 | C | G | -0.1534 | 0.03335 | 4.36E-06 |  | 29,672 | 357,772 | 0.0156794 | 0.0182692 | 0.390759 |
| 9 | rs792792 | T | C | -0.1949 | 0.03989 | 1.07E-06 |  | 29,672 | 357,772 | -0.0125683 | 0.0181435 | 0.488488 |
| 10 | rs8089264 | T | C | 0.2949 | 0.05956 | 7.69E-07 |  | 29,672 | 357,772 | -0.0172206 | 0.0162034 | 0.287885 |
| 11 | rs9400058 | G | A | -0.432 | 0.05081 | 2.66E-17 |  | 29,672 | 357,772 | -0.00506127 | 0.0155589 | 0.744955 |
| 12 | rs9479391 | A | T | -0.2277 | 0.04877 | 3.14E-06 |  | 29,672 | 357,772 | -0.00822028 | 0.0121231 | 0.497729 |

SNP, single nucleotide polymorphism; CD, cluster of differentiation; HF, heart failure; EA, effect allele; OA, other allele; SE, standard error.

**Supplementary Table S6** Information of identified SNPs in exposure (CD20 on CD24+ CD27+) and outcomes (HF).

|  |  | | | **Exposure (CD20 on CD24+ CD27+)** | | |  | **Outcome (HF)** | | | | |
| --- | --- | --- | --- | --- | --- | --- | --- | --- | --- | --- | --- | --- |
|  | **SNP** | **EA** | **OA** | **β** | **SE** | ***p* value** |  | **Case** | **Control** | **β** | **SE** | ***p* value** |
| 1 | rs111259171 | G | A | 0.2545 | 0.04474 | 1.38E-08 |  | 29,672 | 357,772 | -0.0336527 | 0.0474658 | 0.478332 |
| 2 | rs11187166 | T | C | 0.1756 | 0.03165 | 3.07E-08 |  | 29,672 | 357,772 | 0.00890622 | 0.0120901 | 0.461334 |
| 3 | rs12196019 | A | C | 0.2387 | 0.04602 | 2.27E-07 |  | 29,672 | 357,772 | 0.034517 | 0.0247717 | 0.163499 |
| 4 | rs12554596 | G | A | -0.1172 | 0.02548 | 4.39E-06 |  | 29,672 | 357,772 | -0.020485 | 0.0141218 | 0.146893 |
| 5 | rs141121779 | A | G | 0.2393 | 0.05016 | 1.91E-06 |  | 29,672 | 357,772 | -0.0396111 | 0.0307156 | 0.197188 |
| 6 | rs149214933 | G | T | -0.782 | 0.1638 | 1.87E-06 |  | 29,672 | 357,772 | -0.029562 | 0.0230296 | 0.199265 |
| 7 | rs149695230 | G | T | 0.1899 | 0.04009 | 2.27E-06 |  | 29,672 | 357,772 | -0.0178356 | 0.0227991 | 0.434041 |
| 8 | rs179212 | C | T | -0.2158 | 0.04605 | 2.88E-06 |  | 29,672 | 357,772 | 0.00816224 | 0.0178446 | 0.64738 |
| 9 | rs2114442 | T | C | 0.1204 | 0.02503 | 1.57E-06 |  | 29,672 | 357,772 | -0.0037684 | 0.00933917 | 0.686577 |
| 10 | rs55809481 | C | T | -0.283 | 0.0286 | 8.18E-23 |  | 29,672 | 357,772 | -0.0168285 | 0.0101755 | 0.0981612 |
| 11 | rs61731791 | T | C | 0.2236 | 0.04215 | 1.20E-07 |  | 29,672 | 357,772 | -0.0121788 | 0.0289562 | 0.674051 |
| 12 | rs62000215 | T | C | -0.8478 | 0.1832 | 3.82E-06 |  | 29,672 | 357,772 | -0.0241732 | 0.0231353 | 0.296086 |
| 13 | rs6459417 | T | C | 0.2138 | 0.03693 | 7.67E-09 |  | 29,672 | 357,772 | 0.0013368 | 0.0148902 | 0.928464 |
| 14 | rs6922309 | C | G | 0.2142 | 0.04001 | 9.16E-08 |  | 29,672 | 357,772 | 0.0253283 | 0.025481 | 0.32022 |
| 15 | rs7128035 | C | T | -0.1306 | 0.02723 | 1.68E-06 |  | 29,672 | 357,772 | -0.00369399 | 0.0108754 | 0.73411 |
| 16 | rs74621542 | C | T | -0.2878 | 0.04216 | 1.03E-11 |  | 29,672 | 357,772 | 0.00721122 | 0.0206136 | 0.726467 |
| 17 | rs9400057 | A | G | 0.256 | 0.05363 | 1.88E-06 |  | 29,672 | 357,772 | 0.0408344 | 0.0272307 | 0.133725 |

SNP, single nucleotide polymorphism; CD, cluster of differentiation; HF, heart failure; EA, effect allele; OA, other allele; SE, standard error.

**Supplementary Table S7** Information of identified SNPs in exposure (CD19 on CD20-) and outcomes (HF).

|  |  | | | **Exposure (CD19 on CD20-)** | | |  | **Outcome (HF)** | | | | |
| --- | --- | --- | --- | --- | --- | --- | --- | --- | --- | --- | --- | --- |
|  | **SNP** | **EA** | **OA** | **β** | **SE** | ***p* value** |  | **Case** | **Control** | **β** | **SE** | ***p* value** |
| 1 | rs11748905 | C | T | -1.009 | 0.1845 | 4.78E-08 |  | 29,672 | 357,772 | -0.0200009 | 0.0320267 | 0.532295 |
| 2 | rs13390788 | G | A | 0.13 | 0.02761 | 2.60E-06 |  | 29,672 | 357,772 | 0.00271186 | 0.0113012 | 0.810359 |
| 3 | rs164020 | T | A | 0.1148 | 0.02445 | 2.77E-06 |  | 29,672 | 357,772 | -0.0109922 | 0.00968106 | 0.256194 |
| 4 | rs183942882 | G | A | -1.714 | 0.3431 | 6.17E-07 |  | 29,672 | 357,772 | -0.081186 | 0.0368173 | 0.0274467 |
| 5 | rs72901497 | G | A | 0.1646 | 0.03249 | 4.27E-07 |  | 29,672 | 357,772 | 0.0147453 | 0.0138954 | 0.288618 |
| 6 | rs7580866 | C | T | 0.113 | 0.02455 | 4.25E-06 |  | 29,672 | 357,772 | 0.00404747 | 0.00935933 | 0.665413 |
| 7 | rs997173 | T | C | -0.1819 | 0.03977 | 4.94E-06 |  | 29,672 | 357,772 | -0.0161235 | 0.0160377 | 0.314728 |

SNP, single nucleotide polymorphism; CD, cluster of differentiation; HF, heart failure; EA, effect allele; OA, other allele; SE, standard error.

**Supplementary Table S8** Information of identified SNPs in exposure (CD62L- CD86+ myeloid DC %DC) and outcomes (HF).

|  |  | | | **Exposure (CD62L- CD86+ myeloid DC %DC)** | | |  | **Outcome (HF)** | | | | |
| --- | --- | --- | --- | --- | --- | --- | --- | --- | --- | --- | --- | --- |
|  | **SNP** | **EA** | **OA** | **β** | **SE** | ***p* value** |  | **Case** | **Control** | **β** | **SE** | ***p* value** |
| 1 | rs113995133 | A | G | -0.6516 | 0.1277 | 3.54E-07 |  | 29,672 | 357,772 | -0.0140669 | 0.0626456 | 0.822332 |
| 2 | rs117690664 | A | C | -2.506 | 0.5056 | 7.49E-07 |  | 29,672 | 357,772 | -0.0779588 | 0.0443335 | 0.0786683 |
| 3 | rs143140559 | T | C | 0.71 | 0.1289 | 3.88E-08 |  | 29,672 | 357,772 | -0.0180992 | 0.0847667 | 0.830923 |
| 4 | rs147678401 | A | G | 0.3215 | 0.06383 | 4.97E-07 |  | 29,672 | 357,772 | 0.038963 | 0.0215622 | 0.070762 |
| 5 | rs2788088 | A | G | -0.1283 | 0.02719 | 2.49E-06 |  | 29,672 | 357,772 | 0.0185449 | 0.0100514 | 0.0650354 |
| 6 | rs4761369 | T | C | 0.1553 | 0.03329 | 3.19E-06 |  | 29,672 | 357,772 | 0.00417451 | 0.0116961 | 0.721156 |
| 7 | rs500809 | A | G | 0.1195 | 0.02597 | 4.38E-06 |  | 29,672 | 357,772 | 0.0145109 | 0.00972688 | 0.135741 |
| 8 | rs6537835 | A | G | -0.1706 | 0.03712 | 4.48E-06 |  | 29,672 | 357,772 | -0.021377 | 0.0125131 | 0.0875669 |
| 9 | rs71632979 | G | A | 0.929 | 0.0268 | 1.00E-200 |  | 29,672 | 357,772 | 0.0202234 | 0.0151355 | 0.181498 |
| 10 | rs71639910 | A | G | 0.4245 | 0.05172 | 3.16E-16 |  | 29,672 | 357,772 | 0.0805259 | 0.0278478 | 0.00383239 |
| 11 | rs77107932 | C | A | -0.4247 | 0.08194 | 2.31E-07 |  | 29,672 | 357,772 | 0.0203311 | 0.0315182 | 0.51889 |

SNP, single nucleotide polymorphism; CD, cluster of differentiation; DC, dendritic cell; HF, heart failure; EA, effect allele; OA, other allele; SE, standard error.

**Supplementary Table S9** Information of identified SNPs in exposure (HLA DR+ CD4+ AC) and outcomes (HF).

|  |  | | | **Exposure (HLA DR+ CD4+ AC)** | | |  | **Outcome (HF)** | | | | |
| --- | --- | --- | --- | --- | --- | --- | --- | --- | --- | --- | --- | --- |
|  | **SNP** | **EA** | **OA** | **β** | **SE** | ***p* value** |  | **Case** | **Control** | **β** | **SE** | ***p* value** |
| 1 | rs12166155 | G | C | 0.1146 | 0.025 | 4.70E-06 |  | 29,672 | 357,772 | 0.00623562 | 0.0096825 | 0.51957 |
| 2 | rs1322067 | G | A | -0.1511 | 0.02575 | 4.75E-09 |  | 29,672 | 357,772 | 0.000113636 | 0.0093299 | 0.990282 |
| 3 | rs17198888 | G | A | -0.2281 | 0.04783 | 1.93E-06 |  | 29,672 | 357,772 | -0.00597801 | 0.0112539 | 0.595285 |
| 4 | rs2064600 | A | G | -0.3548 | 0.07367 | 1.53E-06 |  | 29,672 | 357,772 | -0.0945523 | 0.0331695 | 0.00436405 |
| 5 | rs3087456 | A | G | -0.2926 | 0.02777 | 1.40E-25 |  | 29,672 | 357,772 | -0.00351102 | 0.0105946 | 0.740343 |
| 6 | rs3134968 | G | A | 0.3737 | 0.07884 | 2.23E-06 |  | 29,672 | 357,772 | 0.0210391 | 0.0111598 | 0.0593936 |
| 7 | rs55849757 | T | C | -0.1129 | 0.02435 | 3.69E-06 |  | 29,672 | 357,772 | -0.00846312 | 0.00945323 | 0.370647 |
| 8 | rs6767083 | A | C | 0.1335 | 0.02844 | 2.78E-06 |  | 29,672 | 357,772 | -0.00478021 | 0.0115502 | 0.678974 |
| 9 | rs7150202 | T | A | 0.1473 | 0.03164 | 3.33E-06 |  | 29,672 | 357,772 | -0.00731465 | 0.0129395 | 0.571874 |
| 10 | rs7405251 | G | T | -0.1151 | 0.02473 | 3.35E-06 |  | 29,672 | 357,772 | -0.0128402 | 0.0102465 | 0.210156 |
| 11 | rs78229126 | A | C | 0.1603 | 0.03437 | 3.23E-06 |  | 29,672 | 357,772 | 0.00619309 | 0.0153213 | 0.686055 |
| 12 | rs9613063 | T | C | -0.1553 | 0.03005 | 2.50E-07 |  | 29,672 | 357,772 | 0.00963168 | 0.0132986 | 0.468903 |

SNP, single nucleotide polymorphism; HLA DR, human leukocyte antigen D-related; CD, cluster of differentiation; AC, absolute count; HF, heart failure; EA, effect allele; OA, other allele; SE, standard error.

**Supplementary Table S10** Information of identified SNPs in exposure (EM CD8br AC) and outcomes (HF).

|  |  | | | **Exposure (EM CD8br AC)** | | |  | **Outcome (HF)** | | | | |
| --- | --- | --- | --- | --- | --- | --- | --- | --- | --- | --- | --- | --- |
|  | **SNP** | **EA** | **OA** | **β** | **SE** | ***p* value** |  | **Case** | **Control** | **β** | **SE** | ***p* value** |
| 1 | rs11153123 | G | A | -0.1435 | 0.03074 | 3.15E-06 |  | 29,672 | 357,772 | 0.00347261 | 0.00947287 | 0.713929 |
| 2 | rs11166573 | A | G | -0.1203 | 0.02612 | 4.25E-06 |  | 29,672 | 357,772 | -0.00362583 | 0.00984655 | 0.712699 |
| 3 | rs113985770 | A | G | -0.6238 | 0.133 | 2.85E-06 |  | 29,672 | 357,772 | -0.0437319 | 0.0408064 | 0.283859 |
| 4 | rs1183901 | C | T | -0.1356 | 0.02714 | 6.17E-07 |  | 29,672 | 357,772 | -0.0105131 | 0.00948105 | 0.267495 |
| 5 | rs13187183 | C | T | -0.2346 | 0.04655 | 4.90E-07 |  | 29,672 | 357,772 | 0.0394106 | 0.0185898 | 0.0340056 |
| 6 | rs143237141 | A | G | 0.3181 | 0.06864 | 3.71E-06 |  | 29,672 | 357,772 | 0.00296128 | 0.0212286 | 0.889059 |
| 7 | rs17583875 | A | G | -1.51 | 0.2336 | 1.15E-10 |  | 29,672 | 357,772 | -0.0780307 | 0.0323384 | 0.0158241 |
| 8 | rs2378966 | T | C | 0.1277 | 0.0258 | 7.85E-07 |  | 29,672 | 357,772 | 0.0194789 | 0.00952982 | 0.0409543 |
| 9 | rs3130685 | T | C | 0.1803 | 0.03185 | 1.62E-08 |  | 29,672 | 357,772 | 0.0147572 | 0.00938364 | 0.115799 |
| 10 | rs58905133 | G | A | 0.1375 | 0.02754 | 6.19E-07 |  | 29,672 | 357,772 | 0.00564883 | 0.00947306 | 0.550971 |
| 11 | rs593435 | T | C | 0.1235 | 0.02599 | 2.11E-06 |  | 29,672 | 357,772 | 0.00934198 | 0.00937807 | 0.319176 |
| 12 | rs73195098 | A | C | 0.373 | 0.0791 | 2.51E-06 |  | 29,672 | 357,772 | 0.048312 | 0.028016 | 0.0846272 |
| 13 | rs76668354 | G | C | -0.7049 | 0.1373 | 2.97E-07 |  | 29,672 | 357,772 | -0.0191645 | 0.0195502 | 0.326953 |
| 14 | rs9382165 | G | A | 0.236 | 0.04837 | 1.11E-06 |  | 29,672 | 357,772 | 0.0234391 | 0.014656 | 0.109757 |

SNP, single nucleotide polymorphism; EM, effector memory; CD, cluster of differentiation; AC, absolute count; HF, heart failure; EA, effect allele; OA, other allele; SE, standard error.

**Supplementary Table S11** The results of MR-Egger intercept analysis.

| **Exposure** | **Outcome** | **Egger_intercept** | **SE** | ***p* value** |
| --- | --- | --- | --- | --- |
| CD66b++ myeloid cell AC | HF | 0.010835502 | 0.010971446 | 0.37925361 |
| HLA DR on CD14- CD16+ monocyte | HF | 0.001409099 | 0.008303895 | 0.869005927 |
| IgD on unsw mem | HF | 0.014249619 | 0.007661933 | 0.087593563 |
| CD4 on CD4+ | HF | 0.007492577 | 0.00787129 | 0.369023947 |
| CD24 on IgD+ CD38- | HF | 0.000234448 | 0.006373026 | 0.971378376 |
| CD20 on CD24+ CD27+ | HF | -0.001346885 | 0.006814318 | 0.845968512 |
| CD19 on CD20- | HF | -0.003749326 | 0.006121034 | 0.566970011 |
| CD62L- CD86+ myeloid DC %DC | HF | 0.001988392 | 0.008433132 | 0.81887879 |
| HLA DR+ CD4+ AC | HF | -0.005577562 | 0.0081564 | 0.509619614 |
| EM CD8br AC | HF | 0.001637614 | 0.005246156 | 0.760280164 |

MR, Mendelian randomization; CD, cluster of differentiation; AC, absolute count; HLA DR, human leukocyte antigen D-related; DC, dendritic cell; EM, effector memory; HF, heart failure; SE, standard error.

**Supplementary Table S12** The results of Cochran's Q analysis.

| **Exposure** | **Outcome** | **Method** | **Q** | **Q_df** | **Q_*p* val** |
| --- | --- | --- | --- | --- | --- |
| CD66b++ myeloid cell AC | HF | MR Egger | 1.863323052 | 4 | 0.760878951 |
| CD66b++ myeloid cell AC | HF | Inverse variance weighted | 2.838695204 | 5 | 0.724837401 |
| HLA DR on CD14- CD16+ monocyte | HF | MR Egger | 9.161058678 | 9 | 0.422541965 |
| HLA DR on CD14- CD16+ monocyte | HF | Inverse variance weighted | 9.190369113 | 10 | 0.514137313 |
| IgD on unsw mem | HF | MR Egger | 13.28263439 | 12 | 0.348836733 |
| IgD on unsw mem | HF | Inverse variance weighted | 17.11117045 | 13 | 0.194271724 |
| CD4 on CD4+ | HF | MR Egger | 7.854372018 | 8 | 0.447823412 |
| CD4 on CD4+ | HF | Inverse variance weighted | 8.760460429 | 9 | 0.459673305 |
| CD24 on IgD+ CD38- | HF | MR Egger | 11.87155701 | 10 | 0.293744715 |
| CD24 on IgD+ CD38- | HF | Inverse variance weighted | 11.87316362 | 11 | 0.373252292 |
| CD20 on CD24+ CD27+ | HF | MR Egger | 12.05693151 | 15 | 0.674714753 |
| CD20 on CD24+ CD27+ | HF | Inverse variance weighted | 12.09599904 | 16 | 0.737345322 |
| CD19 on CD20- | HF | MR Egger | 3.393714944 | 5 | 0.639527328 |
| CD19 on CD20- | HF | Inverse variance weighted | 3.768909666 | 6 | 0.707915293 |
| CD62L- CD86+ myeloid DC %DC | HF | MR Egger | 17.14666375 | 9 | 0.046468165 |
| CD62L- CD86+ myeloid DC %DC | HF | Inverse variance weighted | 17.25258018 | 10 | 0.068958543 |
| HLA DR+ CD4+ AC | HF | MR Egger | 10.80335971 | 10 | 0.37304202 |
| HLA DR+ CD4+ AC | HF | Inverse variance weighted | 11.30854487 | 11 | 0.417788145 |
| EM CD8br AC | HF | MR Egger | 14.15859061 | 12 | 0.290695863 |
| EM CD8br AC | HF | Inverse variance weighted | 14.27355925 | 13 | 0.354864626 |

CD, cluster of differentiation; AC, absolute count; IVW, inverse variance weighted; HLA DR, human leukocyte antigen D-related; DC, dendritic cell; EM, effector memory; HF, heart failure; MR, Mendelian randomization.


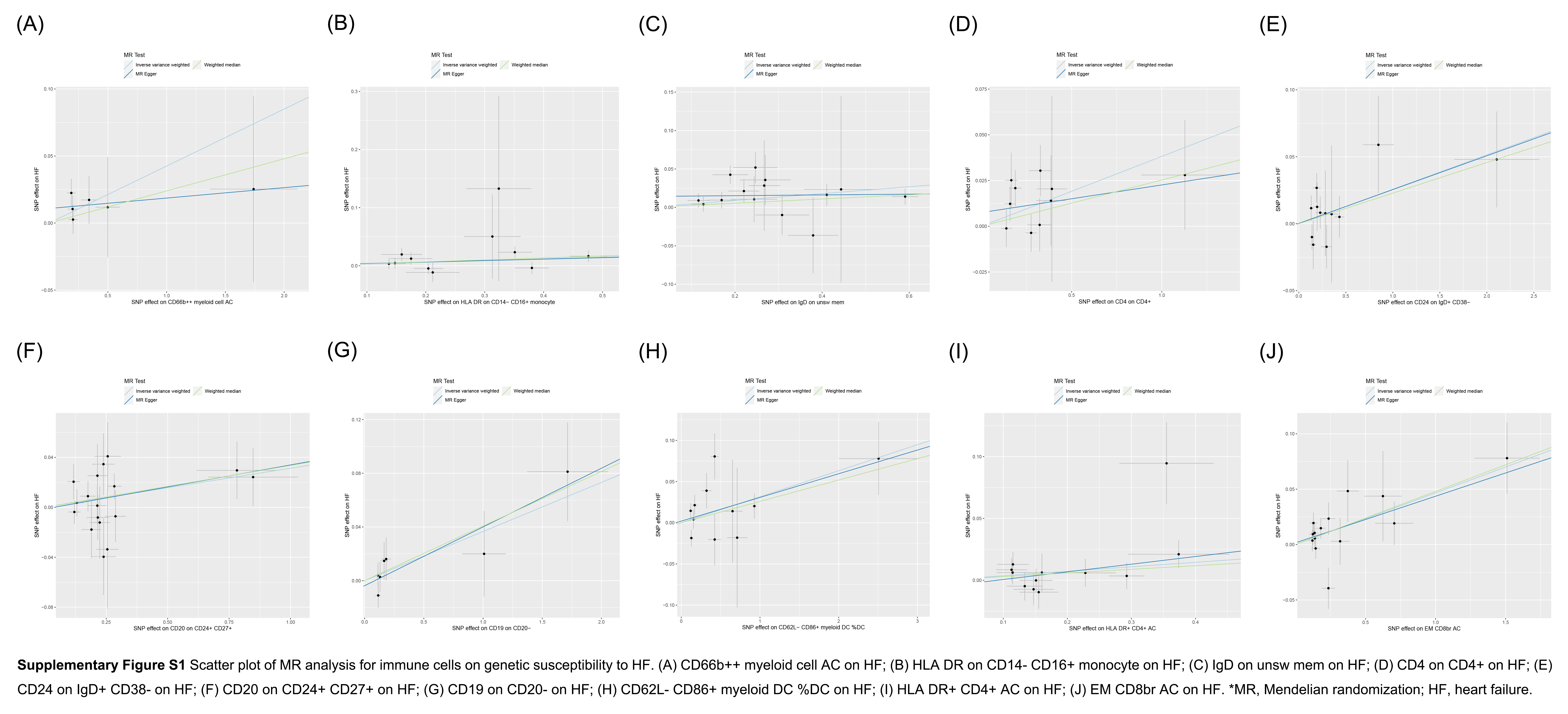


**Supplementary Figure S1** Scatter plot of MR analysis for immune cells on genetic susceptibility to HF. (A) CD66b++ myeloid cell AC on HF; (B) HLA DR on CD14- CD16+ monocyte on HF; (C) IgD on unsw mem on HF; (D) CD4 on CD4+ on HF; (E)CD24 on IgD+ CD38- on HF; (F) CD20 on CD24+ CD27+ on HF; (G) CD19 on CD20- on HF; (H) CD62L- CD86+ myeloid DC %DC on HF; (I) HLA DR+ CD4+ AC on HF; (J) EM CD8br AC on HF. MR, Mendelian randomization; HF, heart failure; CD, cluster of differentiation; AC, absolute count; HLA DR, human leukocyte antigen D-related; DC, dendritic cell; EM, effector memory.


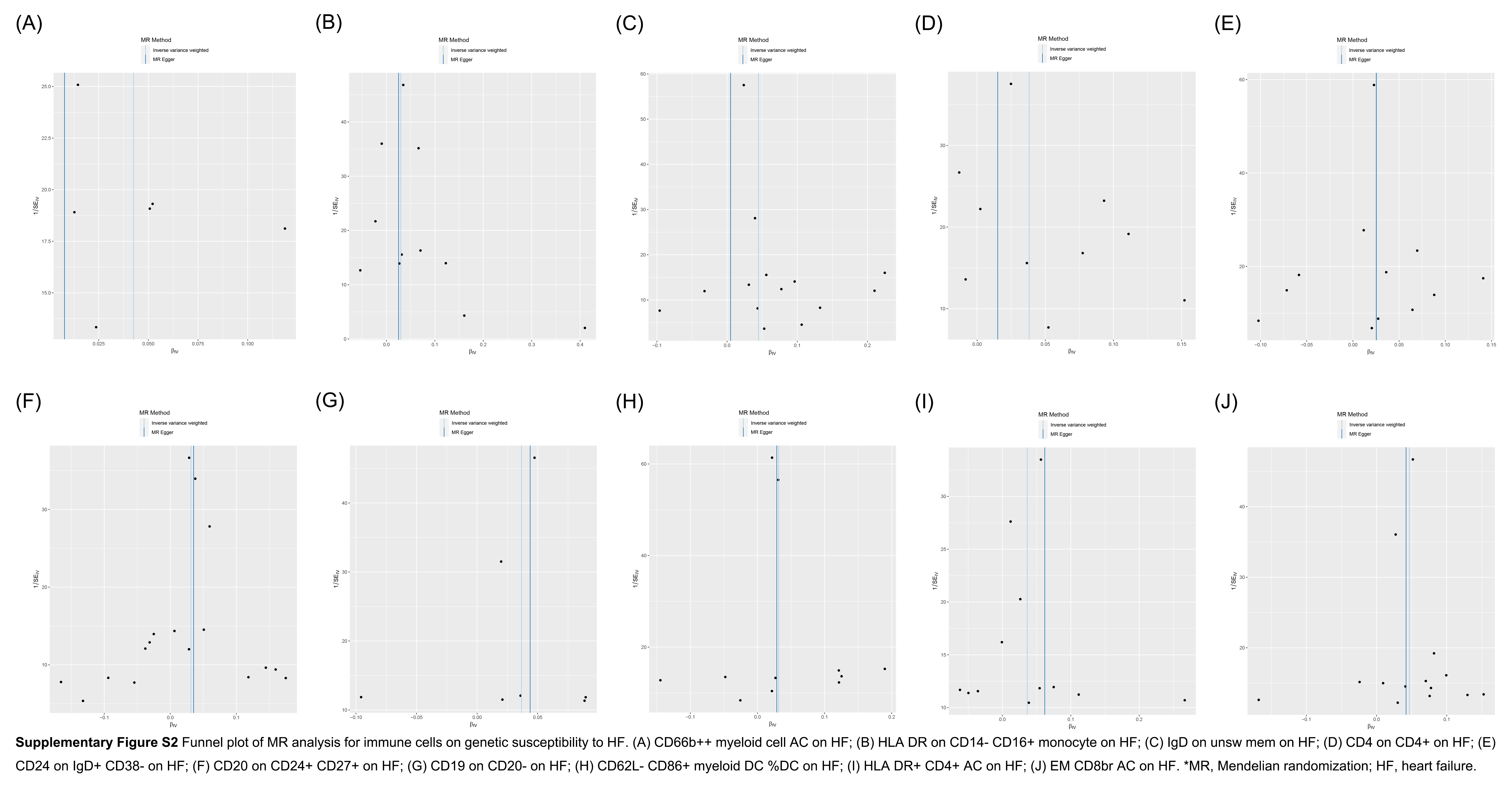


**Supplementary Figure S2** Funnel plot of MR analysis for immune cells on genetic susceptibility to HF. (A) CD66b++ myeloid cell AC on HF; (B) HLA DR on CD14- CD16+ monocyte on HF; (C) IgD on unsw mem on HF; (D) CD4 on CD4+ on HF; (E)CD24 on IgD+ CD38- on HF; (F) CD20 on CD24+ CD27+ on HF; (G) CD19 on CD20- on HF; (H) CD62L- CD86+ myeloid DC %DC on HF; (I) HLA DR+ CD4+ AC on HF; (J) EM CD8br AC on HF. MR, Mendelian randomization; HF, heart failure; CD, cluster of differentiation; AC, absolute count; HLA DR, human leukocyte antigen D-related; DC, dendritic cell; EM, effector memory.


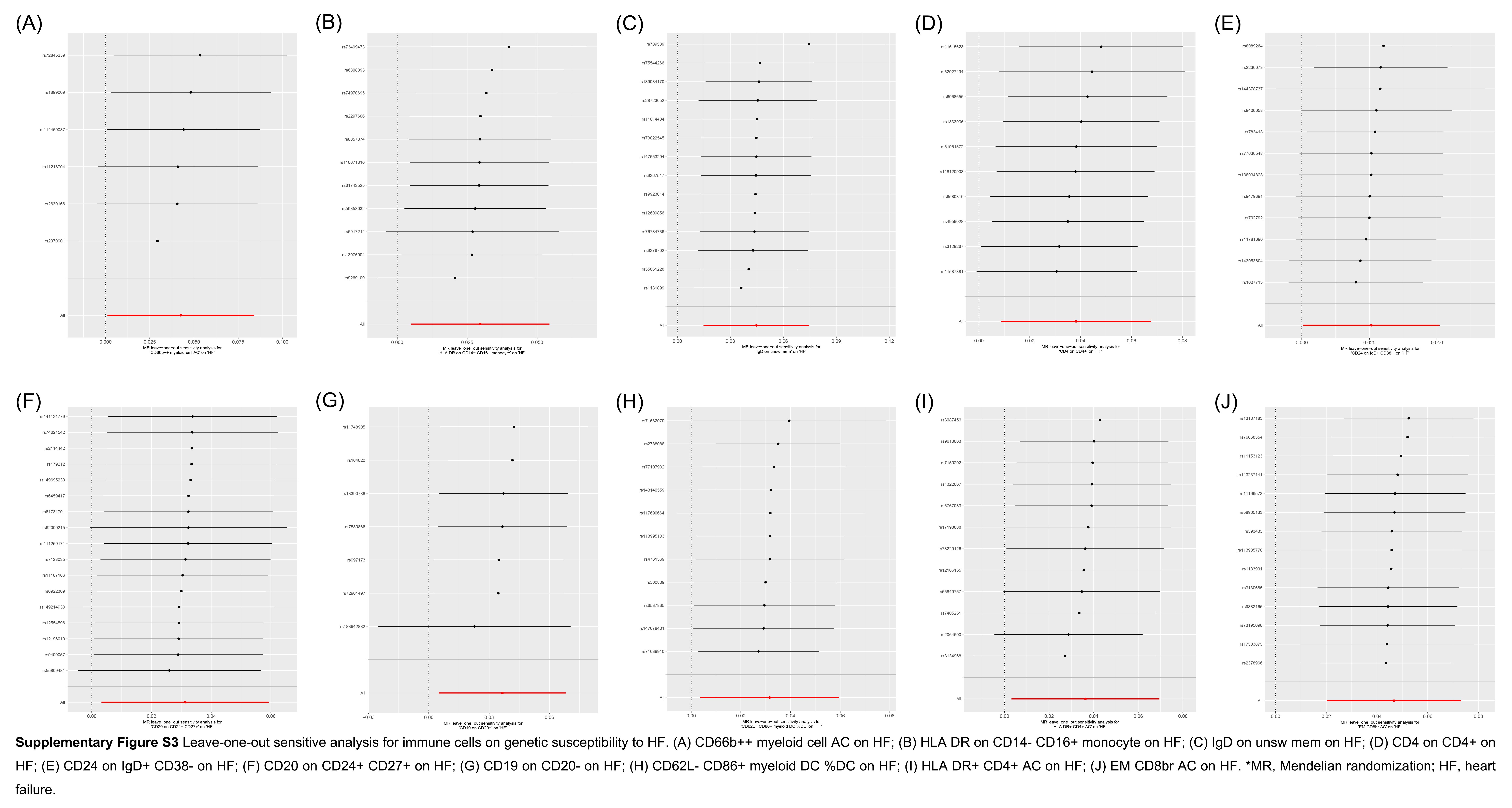


**Supplementary Figure S3** Leave-one-out sensitive analysis for immune cells on genetic susceptibility to HF. (A) CD66b++ myeloid cell AC on HF; (B) HLA DR on CD14- CD16+ monocyte on HF; (C) IgD on unsw mem on HF; (D) CD4 on CD4+ on HF; (E) CD24 on IgD+ CD38- on HF; (F) CD20 on CD24+ CD27+ on HF; (G) CD19 on CD20- on HF; (H) CD62L- CD86+ myeloid DC %DC on HF; (I) HLA DR+ CD4+ AC on HF; (J) EM CD8br AC on HF. HF, heart failure; MR, Mendelian randomization; CD, cluster of differentiation; AC, absolute count; HLA DR, human leukocyte antigen D-related; DC, dendritic cell; EM, effector memory.
